# Regularized Linear Discriminant Analysis of EEG Features in Dementia Patients

**DOI:** 10.3389/fnagi.2016.00273

**Published:** 2016-11-30

**Authors:** Emanuel Neto, Felix Biessmann, Harald Aurlien, Helge Nordby, Tom Eichele

**Affiliations:** ^1^Section for Clinical Neurophysiology, Haukeland University HospitalBergen, Norway; ^2^Institute of Biological and Medical Psychology, University of BergenBergen, Norway; ^3^Amazon Development Center GermanyBerlin, Germany; ^4^K.G. Jebsen Center for Neuropsychiatric DisordersBergen, Norway

**Keywords:** Alzheimer’s disease, vascular dementia, electroencephalogram, qEEG, quantitative analysis, spectral features, group classification, LDA

## Abstract

The present study explores if EEG spectral parameters can discriminate between healthy elderly controls (HC), Alzheimer’s disease (AD) and vascular dementia (VaD) using. We considered EEG data recorded during normal clinical routine with 114 healthy controls (HC), 114 AD, and 114 VaD patients. The spectral features extracted from the EEG were the absolute delta power, decay from lower to higher frequencies, amplitude, center and dispersion of the alpha power and baseline power of the entire frequency spectrum. For discrimination, we submitted these EEG features to regularized linear discriminant analysis algorithm with a 10-fold cross-validation. To check the consistency of the results obtained by our classifiers, we applied bootstrap statistics. Four binary classifiers were used to discriminate HC from AD, HC from VaD, AD from VaD, and HC from dementia patients (AD or VaD). For each model, we measured the discrimination performance using the area under curve (AUC) and the accuracy of the cross-validation (cv-ACC). We applied this procedure using two different sets of predictors. The first set considered all the features extracted from the 22 channels. For the second set of features, we automatically rejected features poorly correlated with their labels. Fairly good results were obtained when discriminating HC from dementia patients with AD or VaD (AUC = 0.84). We also obtained AUC = 0.74 for discrimination of AD from HC, AUC = 0.77 for discrimination of VaD from HC, and finally AUC = 0.61 for discrimination of AD from VaD. Our models were able to separate HC from dementia patients, and also and to discriminate AD from VaD above chance. Our results suggest that these features may be relevant for the clinical assessment of patients with dementia.

## Introduction

Alzheimer’s disease is the most common form of dementia among the elderly population (Jeong, 2004; Kandimalla et al., 2011, 2013, 2014). The second most common form of dementia is VaD (Roman, 2002), affecting approximately 20% of all dementia cases worldwide (Dubois and Hebert, 2001). Presently, there are no specific *in vivo* tests for VaD and AD.

The clinical assessment of dementia is grounded in guidelines from the World Health Organization’s ICD and the American Psychiatric Association’s DSM-IV-TR. These guidelines have been criticized for their lack of specificity (Reilly et al., 2010; McKhann et al., 2011). If fact, many intruments are available to screen dementia, such as CSF measures, CT, MRI examinations, EEG, ultrasound, PET, as well as combination of neurological and psychological tests and laboratory blood analysis. However, many of these examinations are expensive, partially invasive or demand large resources. Furthermore, at early stages these dementias present several symptomatic similarities, creating diagnostic uncertainty (Gearing et al., 1995; Massoud et al., 1999). Consequently, finding alternative methods to detect dementia and classify subtypes is a relevant research topic.

### EEG Based Dementia Diagnosis

Electroencephalogram is a widely available and non-invasive instrument (Rossini et al., 2007). The clinical assessment of an EEG is based on the visual expert interpretation of patient’s electrophisiological activity in a spatio-temporal scale. Classical EEG biomarkers such as relative power and dominant activity rythms in conventional frequency bands and at specific brain regions were shown to be valuable measures to screen dementia (Dierks et al., 1995; Signorino et al., 1996; Jelic et al., 1998). Additionally, the literature has shown that spectral analysis of EEG may provide alternative markers to distinguish AD or VaD patients (Signorino et al., 1996; Besthorn et al., 1997; Bonanni et al., 2008). The spectra of VaD patients show increased power in delta and theta frequencies and decreased power in alpha and beta frequencies (Tsuno et al., 2004; Lou et al., 2011; van Straaten et al., 2012). This phenomenon has also been described by other studies and is associated with general cognitive decline (Dierks et al., 1995; Kwak, 2006; Babiloni et al., 2011b; Fraga et al., 2013). AD and VaD patients are commonly described as having reduced frequency of the posterior dominant rhythm compared with healthy subjects (Babiloni et al., 2004; Gawel et al., 2009). When comparing AD with VaD patients, the literature reports two core electrophysiological differences between these two groups: AD patients have lower posterior alpha power when compared with VaD; the lower frequencies power appears to be higher in VaD compared to AD (Signorino et al., 1995, 1996; Babiloni et al., 2004, 2011a).

### Extracting Features from EEG Signals

There are many EEG features that one could potentially use for discrimination purposes. FFT and power spectral density (PSD) are two of the most widely used transforms that allow to extract potential markers such as frequency, power, coherence across the delta, theta, alpha, or beta bands (Dierks et al., 1991; Kwak, 2006; Fraga et al., 2013). However, the analysis of multi-channel EEG data results in high-dimensional data vectors including spatiotemporal information creating a high computational requirement for solving discrimination problems. Another important aspect is the contamination of EEG with muscular or ocular artifacts such as eye movement and eye blinks (Hillyard and Galambos, 1970; Nunez and Srinivasan, 2006). Artifacts are ubiquitous during the EEG recording and contribute to the non-stationarity of EEG signals (Guger et al., 2000; Shenoy et al., 2006). Non-stationarity of the EEG (von Bunau et al., 2009) constitutes one of the major challenges for data analysis and machine learning classification methods (Kaplan et al., 2005)

### Classification Algorithms

Many different classification algorithms are available. In dementia, it has been shown that it is possible to discriminate patients with different levels of cognitive decline through the use of linear and non-linear classification algorithms (Cichocki et al., 2005; Lopez et al., 2009; Elgendi et al., 2011; Gallego-Jutgla et al., 2012; Gutman et al., 2013). Lehmann et al. (2007) tested several algorithms commonly used for identifying AD patients. Specifically, they employed PC LDA, PLS LDA, PC LR, PLS LR, bagging, random forests, SVM, and others, to identify dementia patients. This study concluded that such techniques may indeed provide remarkable performances (accuracies of 70–90%) for clinical diagnosis. However, they emphasized that the classification models should not be trained with small sample size when compared to their feature dimensionality, as this may result in overfitting the model. This problem is often referred in the literature as the curse of dimensionality (Bishop, 1995). In addition, the feature extraction and discrimination methods vary from study to study, making assessment of reproducibility difficult. One classification method that has been widely used in aging studies, specifically to identify AD is LDA (Dierks et al., 1995; Kwak, 2006; Fraga et al., 2013).

#### LDA

The purpose of the LDA classification is to assign observations to the corresponding class based on a set of measurements or predictors by finding an optimal linear transformation that maximizes the class separability (Fisher, 1936). Technically, LDA achieves optimal solutions when predictors or the feature vector is multivariate normally distributed in each group class and when the different group classes have similar covariance (Fisher, 1936). However, due to the non-stationary of EEG signals (Shenoy et al., 2006), this is rarely the case in practice, and models can easily be overfitted, and their predictability overestimated. This is typically the case when EEG data is derived from multi-channels carrying noise captured from adjacent channels, especially if channels are close enough to each other. Whereas this problem could be solved by applying spatial filters that maximize the variance of EEG signals of one class while minimizing the variance from the other class (Ramoser et al., 2000; Blankertz et al., 2008), at the same time, this may result on overfitting the model by changing predictor variances (Reuderink and Poel, 2008; Huang et al., 2010). In addition, large amount of predictors and relatively small sample sizes dramatically induce misclassification errors contributing to overfitting the model. To circumvent this, many classifiers implement feature reduction based on screening and excluding the features that carry the less amount of information regarding the prediction problem. However, this procedure sometimes eliminates significant features from the data affecting the performance of the classification models (Dauwels et al., 2010). Many of these questions have been addressed in a recent review (Haufe et al., 2014) determining the importance of implementing complementary analysis methods to avoid the bias typically found in generalized linear models (GLM) implementations.

#### Regularization with Cross-Validation

The regularization technique is based on replacing the within-group sample covariance by a weighted average of the whole sample covariance using a shrinking intensity parameter (λ). Technically, this parameter increases larger eigenvalues of the covariance matrix while decreasing smaller ones, therefore creating a pooled-covariance matrix that is corrected for the bias when estimating sample-based eigenvalues. The optimal shrinkage parameter is determined by CV (Friedman, 1989; Ye et al., 2006). CV is a technique to estimate the classification error rate by splitting the original sample data into training, CV, and test datasets (Bishop, 2006). The prediction model is calibrated using the training sets, and model parameters are optimized by the CV sets, while the test sets are used primarily for empirical error estimation. An effective approach to address both the sensitivity and dimensionality problems is using regularized LDA (RLDA) with CV denoted in the literature as CV-RLDA. This approach has been used in several studies with high dimensional and non-stationary problems (Goulermas et al., 2005; Maggi et al., 2006; Huang et al., 2008) achieving very high accuracy levels. It has been shown that the choice of the regularization value or technique has great impact on the overall discrimination performance (Lotte and Guan, 2011).

### Aim of this Study

The literature contains a number of EEG studies revealing several biomarkers that are relevant in dementia group differentiation. However, the methodologies of pre-processing and extracting EEG markers are not identical and sample sizes are typically small. In order to understand dementia at a wider level, we believe that the methodologies to extract EEG biomarkers should be unified across studies. Hence, in our former work, we proposed a feature extraction method that reveals six core EEG parameters at channel-level from any standard clinical EEGs (Neto et al., 2015). We have also shown significant differences between AD, VaD, and HC for several of those parameters at specific channels, in accordance with the present literature.

The aim of the present study was to evaluate the relevance of six EEG parameters on differentiating groups of patients with dementia. To assess this, we designed four discrimination models using LDA to distinguish *HC* from *AD, HC* from *VaD, AD* from *VaD* and *HC* from both *AD* and *VaD*. Moreover, we tested the performance of our four models using two different settings of parameters and measured the class separability performance of our models, which we refer to in the methods section.

## Materials and Methods

We applied LDA to generate four binary LDA classifiers on previously extracted EEG features (Neto et al., 2015). In particular, we used six electrophysiological markers that characterize the EEG spectrum and tested them in group discrimination between AD, VaD, and HC using CV-RLDA. In line with other studies, (Besserve et al., 2007; Zhdanov et al., 2007; Shenoy et al., 2008; Velu and de Sa, 2013), the present study used CV-RLDA methodology for group discrimination using six EEG spectral features. We explored differentiation performances between AD and VaD patients by repeatedly rejecting features with low correlation with their corresponding labels.

### Sample

A total of 342 patients were selected for this study, based on the clinical ICD diagnoses. The first group (*n* = 114) was registered as probable AD patients, with ICD codes ICD-10, F00.x or G30.x. The second group (*n* = 114) was patients with probable VaD (*n* = 114) with ICD-10 F01.X. The HC group (*n* = 114) was selected from non-hospitalized individuals, free of any central nervous-system disease or any other brain disease. We controlled for clinical historic record of all subjects and excluded patients whose diagnose had been changed or removed. Only the most recent EEG from each patient was included. All samples were age-gender balanced with age of 72.9 ± 10.5 years and 48% females. We used these datasets to extract spectral features and test their significance for group discrimination. For further details, the reader is referred to Neto et al. (2015).

### Pre-processing

All EEG datasets were acquired using 22 channels positioned in 10–20 system placements (Fp1, Fpz, Fp2, F7, F3, Fz, F4, F8, T3/T7, C3, Cz, C4, T4/T8, T5/P7, P3, Pz, P4, T6/P8, O1, O2, M1, M2), acquired at 128 Hz (*n* = 86), 256 (*n* = 246), and 500 Hz (*n* = 10) using NicoletOne^TM^ EEG system. Input impedances were set to *Z* > 100MΩ. Hardware single pole high-pass (0.16 Hz ± 10%) and low-pass (500 Hz ± 10%) filters were applied with individually to each channel before pre-amplification. EEGs were stored under raw format in the database. All the pre-processing and data analysis was done accordingly to our previous work (Neto et al., 2015), using Mathworks^®^ Matlab environment. EEG raw files were imported to the EEGLab v.10.1.1.0b toolbox (Delorme and Makeig, 2004) using an in-house data-reader. Data were resampled to 256 Hz. From the standard clinical EEG recording protocol that lasts for 20 min and includes eye open/closed conditions, hyperventilation and provocations with photic stimulation, we restricted the input data for analysis to the first 9 min, which contained only the alternating eyes open/closed resting conditions. A 1536-points high-band filter was applied at cut off frequency of 0.5 and a low-pass filter to cut off of 50 Hz using a standard least square linear-phase FIR filter design. EEGs were segmented into non-overlapping epochs of 1 s that were evaluated for possible rejection using automatic amplitude, power, and statistical thresholding. The remaining segments were subjected to an individual independent component analysis (ICA) using the Infomax algorithm with 15 components in order to identify and remove residual contributions from eye movements. The continuous data were reconstructed from the non-artifact components and then segmented into 2 s epochs with 1 s overlap, which is equivalent to the Welch’s procedure (Welch, 1938) with a rectangular windows and 50% segment overlap. Subsequently, the data were transformed into the frequency domain using FFT. Since the frequency spectrum selected for the pre-processing was from 0.5 to 50 Hz, we obtained 100 frequency data points for the 22 channels and a variable number of epochs for each dataset subjected for analysis. The spatial standard deviation (sSTD) index of each epoch was calculated across the 22 channels in the frequency domain according to Lehmann and Skrandies (1980) and *z*-scored. Finally, in order to standardize the amount of data across subjects, we determined the minimum number of existing epochs across subjects that would maximize the inclusion of patients in this study. We considered a total of 334 epochs from each subject (∼5 m 30 s), representing the artifact-free data for 22 channels (sampled at 256 Hz). We then represented each segment by its equivalent frequency domain data and further used them on the feature extraction method.

### Feature Extraction and Feature Selection

We used a fit-curve model (Neto et al., 2015) which enables us to represent the spectra with six parameters ranging from 0.5 to 30 Hz (*S, k, A, c, w*, and *b*). For each spectrum, *S* represents low frequency power (delta), *k* indicates the 1/f decay from lower to higher frequencies where larger values of *k* denote a faster drop-off in power. Parameters *A, c*, and *w* relate to the amplitude, center and dispersion of the alpha power, respectively. Parameter *b* represents a global offset or *baseline* power of the entire frequency spectrum. We then applied the curve-fit model to the average spectral curve of each channel. Each final dataset was therefore represented by a total 132 parameters, which correspond to the six extracted spectral features from twenty-two channels.

### Discrimination Analysis

**FIGURE 1:**
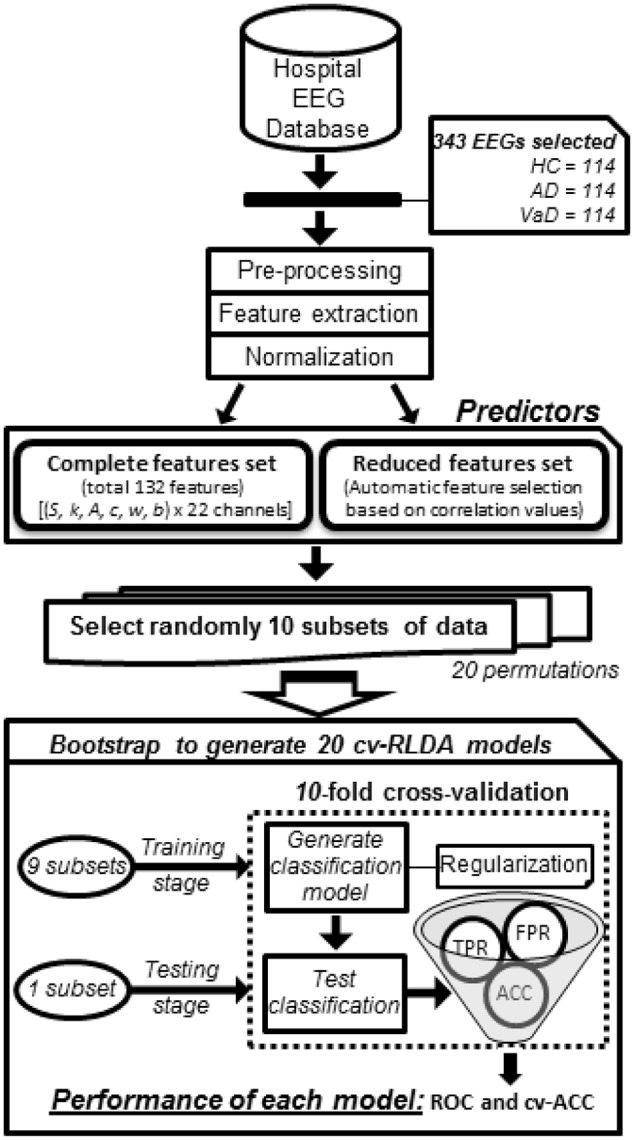
**Workflow diagram of how each group classifier was set up using cv-RLDA.** Spectral features extracted from the EEG of 114 HC, 114 probable AD, and 114 vaD patients were set up as predictors to train and test 20 cv-RLDA models. Values of accuracy (ACC), TPR, and FPR were determined from each fold of CV. Performance values of receiver operating characteristic (ROC) were determined from ratio of TPR and FPR values, and ACC, respectively, averaged over 10-fold. The final values of performance reported for each classifier regard the average of ROC and cv-ACC values obtained from 20 cv-RLDA models.

To test the potential of such features in group discrimination, we tested two different feature sets to generate four binary classifiers which discriminated the classes HC *versus* AD *(Model 1)*, HC *versus* VaD *(Model 2)*, AD *versus* VaD *(Model 3)*, and HC *versus* all dementia patients AD or VaD *(Model 4)* as illustrated in **Figure 1**. These were all implemented by using 10-fold cv-RLDA. The two different set of features are referred as *Complete set of Features* and *Reduced set of Features*. The *Complete Set of Features* comprised all available parameters that were extracted from our fit-curve feature extraction procedure for the 22 channels of each dataset resulting on a total of 132 model predictors. The Reduced set of features is a sub-set of all 132 features. However, we decided to remove features that were poorly correlated with their labels. To determine this, before training the linear classifiers, we computed the correlation of each feature with the target variable, we tested the correlation of each feature with their correspondent labels. We only considered data points in the respective training data set of each particular CV fold. The output of this test is a vector with correlation values (*r*) and their correspondent *p*-value (*p*). The exclusion criteria was to reject features with significantly low correlation index at |*r*| < 0.15 and *p* < 0.01 (two tailed *t*-test). The procedure of excluding features is highly dependent to which datasets are in use when generating each model. Since, we used different sets of data to train each model, we also obtained different values of correlation and *p*-values. Hence, a variable number of features were rejected. The average numbers of used Reduced Set of Features were 74, 68, 18, and 76 for *Model 1, Model 2, Model 3*, and *Model 4*, respectively.

#### cv-RLDA Classifier Models

In order to use the extracted spectral features as predictors on our LDA models, we verified the fundamental assumptions of the LDA (Worth and Cronin, 2003). All predictors met the criteria of being scalar and positive. To transform the predictor matrix into an approximately normal distribution, we applied a log-transformation using box-cox transformation (Box and Cox, 1964) and measured the average, standard deviation and covariance values for the predictors on each group, which we reported as supplementary material showing the normal probability distribution of the predictors before and after the normalization process (Supplementary Table S1; Supplementary Figures S2–S5).

The predictor matrix for each model was constituted by 228 rows that referred to the subjects from each test and a variable number of columns, (132 or 29) that corresponded to the total predictors used in the *Total* or *Reduced Set of Features*, respectively.

To evaluate the linear classification model, we used 10-fold CV. We divided our 228 samples into 10 randomized subsets and determined a fold size of 22. For each fold of the CV, we considered nine subsets for the training and one subset for the testing phases. Therefore, 198 and 22 different datasets were considered on each fold as the training and testing sample, respectively. To set the regularization threshold constant of the regularized LDA model, we used the analytical shrinkage estimator (Ledoit and Wolf, 2004). We alternatively tried nested CV, but found the analytical estimator to yield slightly better results. The permutation order of the training sample may have an impact on the result of the *k*-fold CV (Triba et al., 2015). Therefore, to further validate the robustness of our model, we used bootstrap and generated each model 20 times, each one with the same procedure as described previously, but with a different random selection of data points.

#### Performance Tests

**FIGURE 2:**
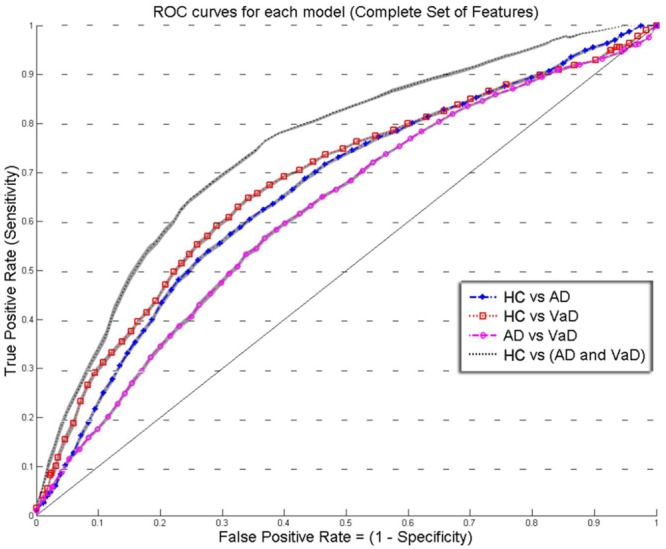
**Overlay of ROC performance curves from each model using the *Complete set of feature* (*S, k, A, c, w*, and *b*).** The shadow plot display the SEM values for each curve. Healthy controls (HC); probable Alzheimer’s disease (AD); vascular dementia (VaD).

**FIGURE 3:**
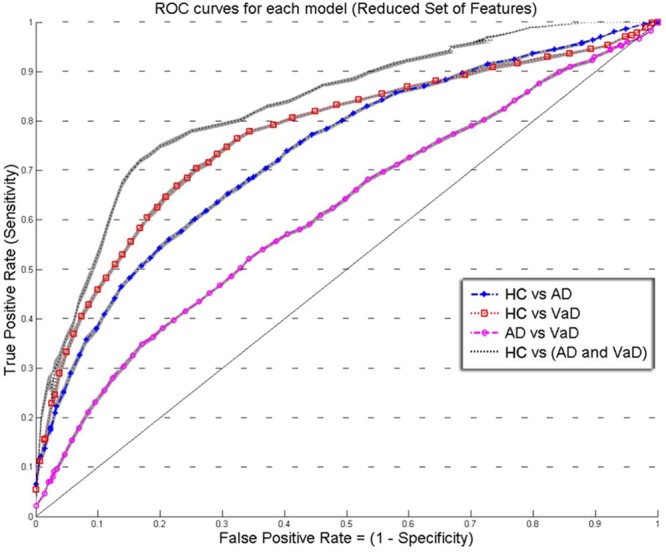
**Overlay of ROC performance curves from each model using the *Reduced set of feature* (*S, A, c*, and *w*).** The shadow plot display the SEM values for each curve. Healthy controls (HC); probable Alzheimer’s disease (AD); vascular dementia (VaD).

To benchmark the performance of our final models, we estimated the average of TPR and FPR from each group classifier. Using these metrics, we determined the ROC curve to represent the classification performance of each model (**Figures 2** and **3**). The ROC displays the trade between the sensitivity and specificity of classification from each model. The AUC for each group classifier provided a measure of the discrimination power which has been used as a gold standard diagnostic marker (Zhou et al., 2011). In addition, we determined the accuracies of the models measuring the CV accuracy. Each model’s cv-ACC was estimated by averaging the accuracies obtained on each 10-fold classification tests. To avoid bias from the order of datasets used during the calibration stage, we iterated 20 times the generation of each of the four models using bootstrap of the *test* sample. For each iteration, a different permutation of the training and testing datasets were chosen obtaining twenty different CV accuracies (cv-ACC) and 20 different areas under curve (AUC) for each model. The final performance results of each model were determined by averaging the twenty cv-ACC and AUC results obtained from each group classifier (**Table 1**).

**Table 1:** Performance values for each final classifier model.

	Complete set of features		Reduced set of features	
Classifier performances	cv-ACC	AUC	cv-ACC	AUC
Model 1 (HC vs. AD)	0.62	0.66	0.67	0.74
Model 2 (HC vs. VaD)	0.65	0.68	0.72	0.77
Model 3 (AD vs. VaD)	0.59	0.62	0.57	0.61
Model 4 (HC vs. AD&VaD)	0.70	0.75	0.77	0.83

*Average of the CV accuracy (cv-ACC) and AUC performed for each final classifier using two different sets of features. The *Complete Set of Features* included a total of 132 predictors and the *Reduced Set of Features* included variable number of predictors based on automatic feature reduction. Values from [0.5–0.6] = *Fail*; [0.6–0.7] = *Poor*; [0.7–0.8] = *Fair*; [0.8–0.9] = *Good*; [0.9–1.0] = *Excellent*.*

## Results

We calculated the *R*^2^ measure for each fit, reflecting the fraction of data variance captured by curve approximation. Additionally, we computed the correlations between model parameters to check for dependence between parameters, which can indicate model redundancies or instabilities in the fitting procedure. Histograms of the *R*^2^ values were presented in Supplementary Figure S1 for each group, and show excellent model fits in nearly all cases. The median goodness-of-fit was 0.91 and the first and third quartiles were 0.92 and 0.98, respectively. We performed a Kruskal–Wallis one-way ANOVA test and no group differences were found in the *R*^2^ values between the groups (*p* < 5.15e^-11^), meaning that the model performed equally well fitting the datasets from each group. Only relatively weak correlations (|*r*|≤ 0.4) between the parameters were found, with the exception of a high correlation between parameters *k* and *b* (*r* = 0.75). This correlation was equally present within each group: *r* = 0.80 for HC, *r* = 0.68 for AD, and *r* = 0.78 for VaD.

**Figures 2** and **3** show an overlay of the receiver operating characteristic (ROC) curves obtained for each group classifier using the Complete Set of Features and Reduced Set of Features.

The FPR and TPR results obtained from each group classifier were averaged across the 20 correspondent model’s results with mean and corresponding SEM values (**Figures 2** and **3**). We found no significant differences of classification (cv-ACC and AUC) for the different permutations of subsets.

When using the Complete Set of Features, the first three RLDA models, *HC* vs *VaD, HC* vs *AD*, and *AD* vs *VaD*, achieved *poor* discrimination performance with AUC of 0.66, 0.66, and 0.62, respectively. The fourth model attained *fair* discrimination for *HC* from *AD* or *VaD* patients with AUC = 0.75. **Figure 2** displays an overlay of all four ROC curves with SEM values from each classifier for the *Complete Set of Features*.

When using the Reduced Set of Features, the first two models, *HC* vs *VaD, HC* vs *AD*, obtained *fair* discrimination performances with AUC of 0.74 and 0.77, respectively. The third model discriminated poorly *AD* vs *VaD* with AUC = 0.61. The fourth model discriminated good *HC* from *AD* or *VaD* patients with AUC = 0.83. **Figure 3** displays an overlay of all four ROC curves from each classifier for the *Reduced Set of Features.*

The corresponding cv-ACC values of each model and AUC median, first and third quartiles are provided as Supplementary Table S2.

## Discussion

Using features selected from routine EEG recordings and averaged across time for every channel, our results show that HC are separable from dementia patients. Furthermore, we were also able to differentiate AD from VaD above chance. Clinical practitioners may not always have the ability to assess the predominant biomarker values of an EEG recording. Quantitative EEG spectral analysis is an important tool that allows extracting such features. We have demonstrated that the tested features are reasonably robust biomarkers for the discrimination of dementia groups.

The extracted features were normalized across groups and with similar class covariance. Under these conditions, the LDA should have optimal discrimination performance for the set of predictors used. We extended our analysis by using a *Reduced Set of Features* excluding predictors which accounted with small or not statistically significant correlation for their corresponding classes. By removing these predictors, we expected better discrimination performance of our models as dimensionality was reduced. The performance of our classifier models was *Good* or *Fair* but not *Excellent*. Both results of performance (AUC) and accuracy (cv-ACC) of all models were improved when the same models were generated using the *Reduced Set of Features*.

Model 1: The discrimination between HC and AD, was initially determined with AUC = 0.66. As expected when generating the same classification model with the *Reduced set of features*, the performance increased significantly to AUC = 0.74 and the cv-ACC model was also improved from cv-AUC = 0.62 to cv-AUC = 0.67, suggesting that these features contain good discriminatory information. The core features that have consistently been shown to describe AD patients is the decrease in alpha power frequency at posterior brain regions and a general power increase of delta and theta rhythms when compared with HC (Huang et al., 2000; Babiloni et al., 2004; Jeong, 2004; Prichep, 2005). Comparing to this model, similar discrimination performances were achieved by other studies, reporting accuracies ranging from 70 to 80% (Vecchio et al., 2013). Although this discrimination power appears to be reasonable in real clinical setting, these accuracies are not enough to be clinically useful alone. Based on spectroscopy and additional EEG variables, another study was able to discriminate AD from HC with 88% sensitivity and 89% specificity (Rodriguez et al., 1998). In fact, recent studies were able to demonstrate better classification performances (0.87 < AUC < 0.94) (Anghinah et al., 2011; Kanda et al., 2014; Buscema et al., 2015). However, the subjects sample, conditions of the EEG recording (e.g., subject awake, eyes open/closed, occurrence of external stimuli), the recording protocol, data processing and used methods induce complexity in the analysis and may compromise the conclusion of such studies (Dauwels et al., 2010). We believe that the accessibility and low complexity of the tested features (*S, k, A, c, w, b*), provided satisfactory information for the discrimination process and may be further be validated as AD descriptors.

Model 2: The discrimination model for HC versus VaD, showed the same trend with slightly better results using the reduced set of features, obtaining an improvement of AUC from 0.68 to 0.77 and of cv-AUC from 0.65 to 0.72. Studies reported that at specific brain regions, the correlation between the underlying structural changes and the EEG power is the marker that contributes most for the discrimination of VaD patients. (Szelies et al., 1994; Gawel et al., 2009). Nevertheless, it has repeatedly been demonstrated that VaD patients have increased delta power, increased diffused theta power, and decreased alpha rhythm (Signorino et al., 1995; Babiloni et al., 2004; Reed et al., 2004; Moretti et al., 2012). In fact, Moretti et al. (2012) suggested that theta/alpha ratio could be a reliable index for the estimation of the individual extent of CV damage. This model did not perform good or excellent. Hence the clinical utility of our features for the classification of VaD patients is yet unsatisfactory and should be complemented with other biomarkers such as neuroimaging.

Model 3: the discrimination of AD vs VaD, obtained the weakest classification results. In contradistinction to other models, when we used the Reduced Set of Features, this model performed poorly. Only poor performances values were obtained using both set of features with AUC = 0.62 and AUC = 0.61, respectively. The cv-ACC was also low with cv-ACC = 0.59 and 0.57 for each set of predictors, respectively. This is unexpected as one study reported performances of sensitivity 0.64% and specificity of 77% for distinguishing VaD and AD (Walker et al., 2000). Additionally, it has been shown that 97% of VaD patients yielded a spectrum with dominant activity between 6.5 and 12 Hz and only 44% of AD when compared with HC (Signorino et al., 1995). When using the *Complete Set of Features*, our results showed poor performance results and that was due to a high correlation between features *k* and *b* (*r* = 0.75). This correlation was equally present within groups: *r* = 0.80 for HC, *r* = 0.68 for AD, and *r* = 0.78 for VaD. As mentioned above, this added redundancy and overfitted the LDA model, hence achieving low performance values. On the other hand, when we tested the *Reduced Set of Features*, the model appeared under-fitted. Our tested (and highly correlated) features *k* and *b* represent theta and beta bands which have been reported as very relevant for discrimination between AD and VaD (Babiloni et al., 2004). We speculate that in order to achieve better discrimination between these types of dementia our model lacked alternative features that characterize theta independently.

Model 4: The discrimination between HC and both groups of dementia patients (AD&VaD) obtained generally *good* discrimination performance for both conditions using the *Complete Set of Features* and the *Reduced Set of Features* with AUC = 0.75 and AUC = 0.83, respectively. The accuracy of the model was also good, presenting values of cv-ACC = 0.70 and cv-ACC = 0.77 for the *Complete* and *Reduced Set of Features*, respectively. AD and VaD patients may suffer occasionally from similar cognitive impairments that both result in neural degradation. The literature showed that these two diseases have many similarities at several electrophysiological markers such as increased power at lower frequencies, decreased and slower alpha or increase of theta power (Ernst Niedermeyer, 2010). Our previous work has demonstrated spectrum similarities between these groups of patients when compared with matched HC (Neto et al., 2015). The performance of model 4 revealed that the tested features characterized appropriately these spectrum differences. Therefore our results suggest that such features may be relevant for discrimination between dementia and healthy groups.

We obtained nearly the same performances measures for the variant models generated with permuted order of the learning and testing subsets and denoted no significant fluctuation on the models performances (cv-ACC and AUC) for the different permutations of subsets. The stable and low variations values obtained for cv-ACC and AUC across the permutations and the 10-fold CV are indicators of high robustness of the models here obtained.

## Conclusion

The EEG features tested in this study for the discrimination between patients with dementia (*AD* or *VaD*) and HC performed generally well. The discrimination between *AD* and *VaD* showed above chance performance. Discrimination between *AD* from HC or *VaD* from HC was obtained with fair results.

Therefore, we conclude that the tested EEG features hold relevant discriminatory information, and, in combination with other markers and other known dementia diagnostic tools such as neuroimaging, may constitute necessary and valuable information to screen dementia in a clinical setting.

## Author Contributions

EN, study design, data analysis, wrote the article. FB, contributed with guidance and implementation code of discrimination analysis algorithms. HA, planning of this study and co-supervision; Selection of patient’s datasets. HN, planning of this study and co-supervision. TE, planning of this study and supervision.

## Conflict of Interest Statement

The authors declare that the research was conducted in the absence of any commercial or financial relationships that could be construed as a potential conflict of interest.
