# Supplementary material for: Regularized Linear Discriminant Analysis of EEG Features in Dementia Patients
Source: Front Aging Neurosci. 2016 Nov 30;8:273. doi: 10.3389/fnagi.2016.00273 (PMC5127828; doi:10.3389/fnagi.2016.00273)
Supplement: Supplementary file 1 [file Data_Sheet_1.pdf]

Regularized linear discriminant analysis of EEG features in dementia patients

Emanuel Neto[1,2], Felix Biessmann[3], Harald Aurlien[1], Helge Nordby[2], Tom Eichele[1,2,4]

(1) Section for clinical neurophysiology, Haukeland University Hospital, Bergen, Norway

(2) Institute of biological and medical psychology, University of Bergen, Norway

(3) Amazon Development Center Germany, Berlin, Germany

(4) K.G. Jebsen Center for Neuropsychiatric Disorders

Supplementary material

Normalization of predictors matrix

Prior to generate the discrimination models, we normalized the data using box-cox log-transformation. We inspected the distribution of our data within and across groups. *Figures s1-s4* provided here as supplementary material, display the normal probability distribution of the predictors for each corresponding class before and after normalization. Values of mean, standard deviation and covariance were also calculated for each class before and after normalization and summarized in *Table s1*.

The first model, HC *vs AD*, the first class, HC presented an initial distribution of predictors with mean 270.42 ± 51.7 and covariance of 2.7×10^3, while the second class, *AD*, presented a mean of 284.03 ± 51.1 and covariance 2.6×10^3. After normalization HC had a feature average of 1.71 ± 0.37 and covariance of 0.134, while *AD* had 1.77 ± 0.37 and covariance of 0.135.

For the second model, HC *vs VaD*, the first class HC presented an initial distribution of predictors with mean 316.59 ± 66.4 and covariance of 4.4×10^3, while *VaD* had 309.0 ± 63.3 and covariance 4.0×10^3. After normalization HC had a feature average of 1.68 ± 0.41 and covariance of 0.167, while *VaD* had 1.68 ± 0.40 and covariance of 0.156.

The third model, *AD vs VaD, AD* presented an initial distribution of predictors with mean 355.81 ± 66.7 and covariance of 4.6×10^3, while *VaD* had 357.5 ± 72.6 and covariance 5.3×10^3. After normalization *AD* had a feature average of 1.55 ± 0.48 and covariance of 0.233, while *VaD* had 1.57 ± 0.41 and covariance of 0.170.

The fourth model, HC *vs (AD+VaD)*, the first class HC presented an initial distribution of predictors with mean 300.67 ± 52.6 and covariance of 2.8×10^3, while the second class *AD+VaD*

had 302.5 ± 56.7 and covariance 3.2x10^{3}. After normalization HC had a feature average of 1.64 ± 0.40 and covariance of 0.163, while *AD+VaD* had 1.61 ± 0.43 and covariance of 0.183.

		$\bar{x}$		σ		*cov*	
	Classifier	***Class1***	***Class2***	***Class1***	***Class2***	***Class1***	***Class2***
**Before normalization**	**Model 1 (HC vs AD)**	270.42	284.03	51.7	51.1	2.7x10^{3}	2.6x10^{3}
	**Model 2 (HC vs VaD)**	316.59	309.0	66.4	63.3	4.4x10^{3}	4.0x10^{3}
	**Model 3 (AD vs VaD)**	355.81	357.5	66.7	72.6	4.6x10^{3}	5.3x10^{3}
	**Model 4 (HC vs AD&VaD)**	300.67	302.5	52.6	56.7	2.8x10^{3}	3.2x10^{3}
**After normalization**	**Model 1 (HC vs AD)**	1.71	1.77	.37	.37	.134	.135
	**Model 2 (HC vs VaD)**	1.68	1.68	.41	.40	.167	.156
	**Model 3 (AD vs VaD)**	1.55	1.57	.48	.41	.233	.170
	**Model 4 (HC vs AD&VaD)**	1.68	1.68	.41	.40	.167	.156

Table s1: Distribution of the predictors matrix data before and after normalization for each classifier using classes of healthy controls (HC), probable Alzheimer's disease (AD) and vascular dementia (VaD). Values of mean ($\bar{x}$), standard deviation (σ) and covariance (*cov*) for each class of subsets on each classifier model.

		cv-ACC		*AUC*			
	Classifier	$\bar{x}$	σ	$\bar{x}$	***median***	***1st Quantil***	***3rd Quantil***
**Complete set of features**	**Model 1 (HC vs AD)**	0.62	0.017	0.66	0.0044	0.0037	0.0059
	**Model 2 (HC vs VaD)**	0.65	0.017	0.68	0.0040	0.0026	0.0054
	**Model 3 (AD vs VaD)**	0.59	0.016	0.62	0.0041	0.0027	0.0051
	**Model 4 (HC vs AD&VaD)**	0.70	0.021	0.75	0.0036	0.0027	0.0074
**Reduced set of features**	**Model 1 (HC vs AD)**	0.67	0.016	0.74	0.0047	0.0038	0.0058
	**Model 2 (HC vs VaD)**	0.72	0.019	0.77	0.054	0.0032	0.0069
	**Model 3 (AD vs VaD)**	0.57	0.021	0.61	0.0041	0.0034	0.0053
	**Model 4 (HC vs AD&VaD)**	0.77	0.019	0.83	0.0044	0.0036	0.0090

Table s2: Results with performance values (cv-ACC and AUC) for each classifier model using the Complete Set of Features and the Reduced Set of Features. Mean ($\boldsymbol{x}$) and standard deviation ($\boldsymbol{\sigma}$) for the cross-validation accuracy (cv-ACC). Mean ($\boldsymbol{x}$), median, first and third quantile for the area under curve (AUC) measure.

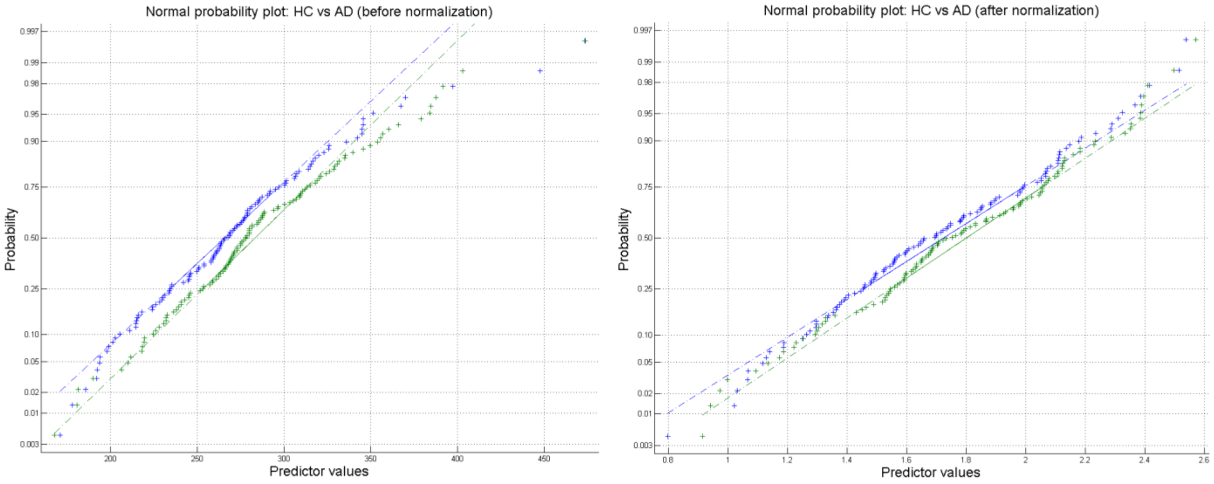

a) b)

Figure s2: Normal probability plots of the predictor's matrix **a)** before normalization and **b)** after normalization for both classes of healthy controls (HC) in blue, and probable Alzheimer's disease (AD) in green. Before normalization HC had a feature average of 270.42 ± 51.7 and covariance of $2.7x10^3$, while *AD* had 284.03 ± 51.1 and covariance $2.6x10^3$. **b)** After normalization HC had a feature average of 1.71 ± 0.37 and covariance of 0.134, while AD had 1.77 ± 0.37 and covariance of 0.135.

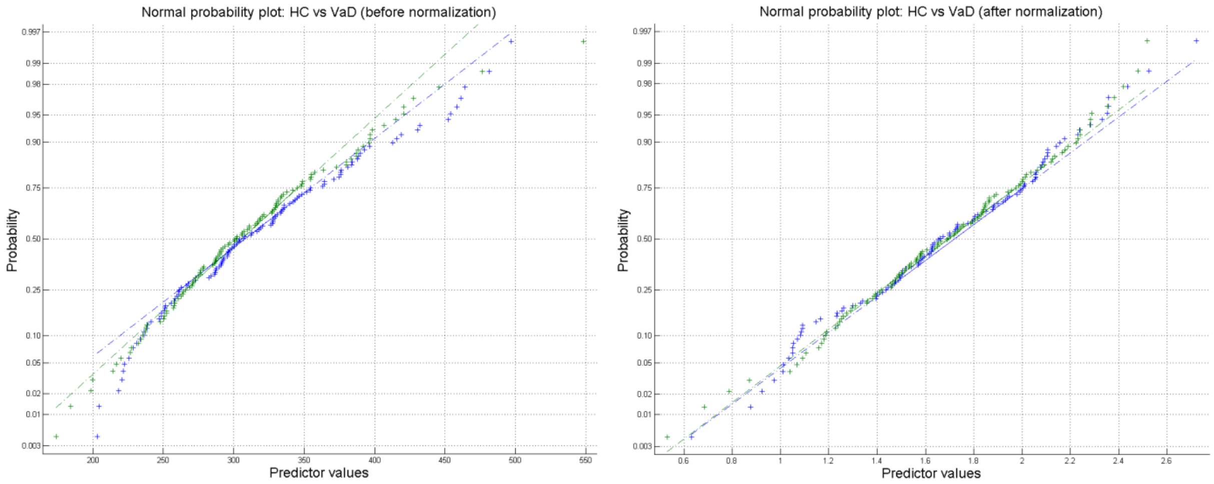

a) b)

Figure s3: Normal probability plots of the predictor's matrix **a)** before normalization and **b)** after normalization for both classes of healthy controls (HC) in blue, and probable vascular dementia (VaD) in green. **a)** Before normalization HC had a feature average of 316.59 ± 66.4 and covariance of $4.4x10^3$, while *VaD* had 309.0 ± 63.3 and covariance $4.0x10^3$. **b)** After normalization HC had a feature average of 1.68 ± 0.41 and covariance of 0.167, while *VaD* had 1.68 ± 0.40 and covariance of 0.156.

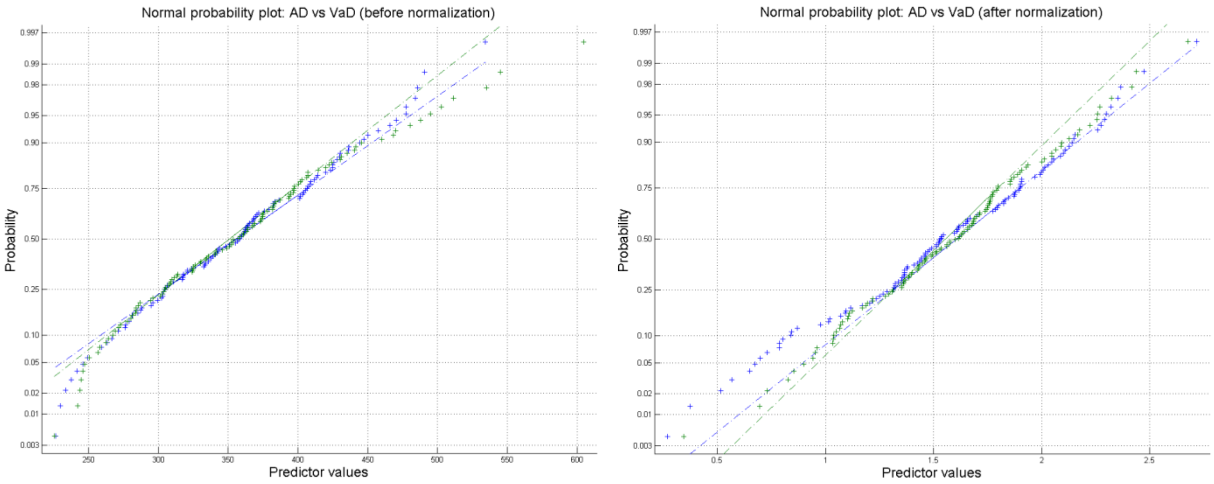

a) b)

Figure s4: Normal probability plots of the predictor's matrix **a)** before normalization and **b)** after normalization for both classes of probable Alzheimer's disease (AD) in blue, and vascular dementia (Vas) in green. **a)** Before normalization *AD* had a feature average of 355.81 ± 66.7 and covariance of $4.6\text{x}10^3$, while *VaD* had 357.5 ± 72.6 and covariance $5.3\text{x}10^3$. **b)** After normalization *AD* had a feature average of 1.55 ± 0.48 and covariance of 0.233, while *VaD* had 1.57 ± 0.41 and covariance of 0.170.

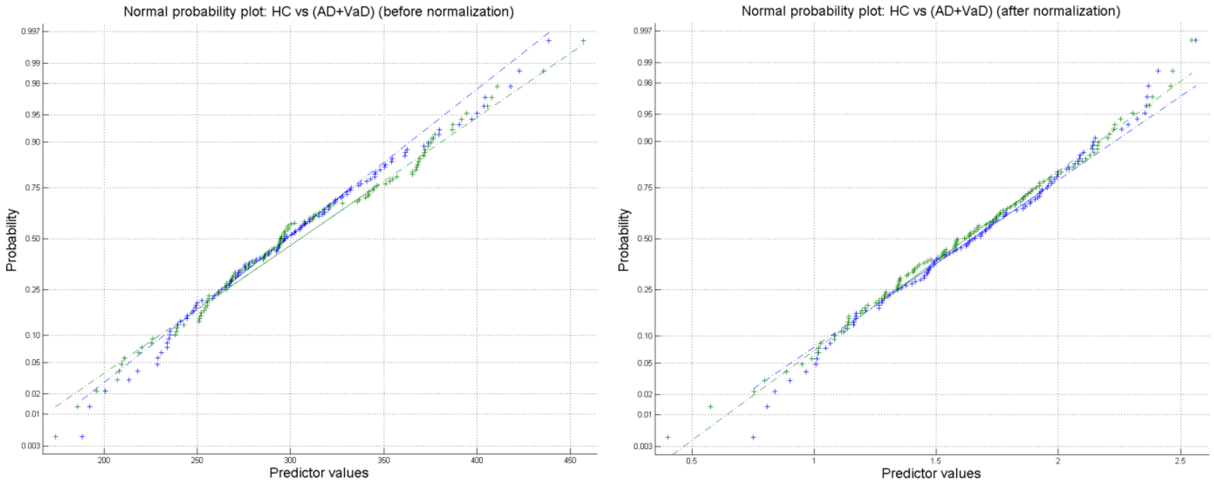

a) b)

Figure s5: Normal probability plots of the predictor's matrix **a)** before normalization and **b)** after normalization for class of healthy controls (HC) in blue, and a second class of both probable Alzheimer's disease (AD) and vascular dementia (vaD) in green. **a)** Before normalization HC had a feature average of 300.67 ± 52.6 and covariance of $2.8\text{x}10^3$, while *AD*&*VaD* had 302.5 ± 56.7 and covariance $3.2\text{x}10^3$. **b)** After normalization HC had a feature average of 1.64 ± 0.40 and covariance of 0.163, while *AD*&*VaD* had 1.61 ± 0.43 and covariance of 0.183.
