# Supplementary figures and images for: Regularized Linear Discriminant Analysis of EEG Features in Dementia Patients
Source: Front Aging Neurosci. 2016 Nov 30;8:273. doi: 10.3389/fnagi.2016.00273 (PMC5127828; doi:10.3389/fnagi.2016.00273)

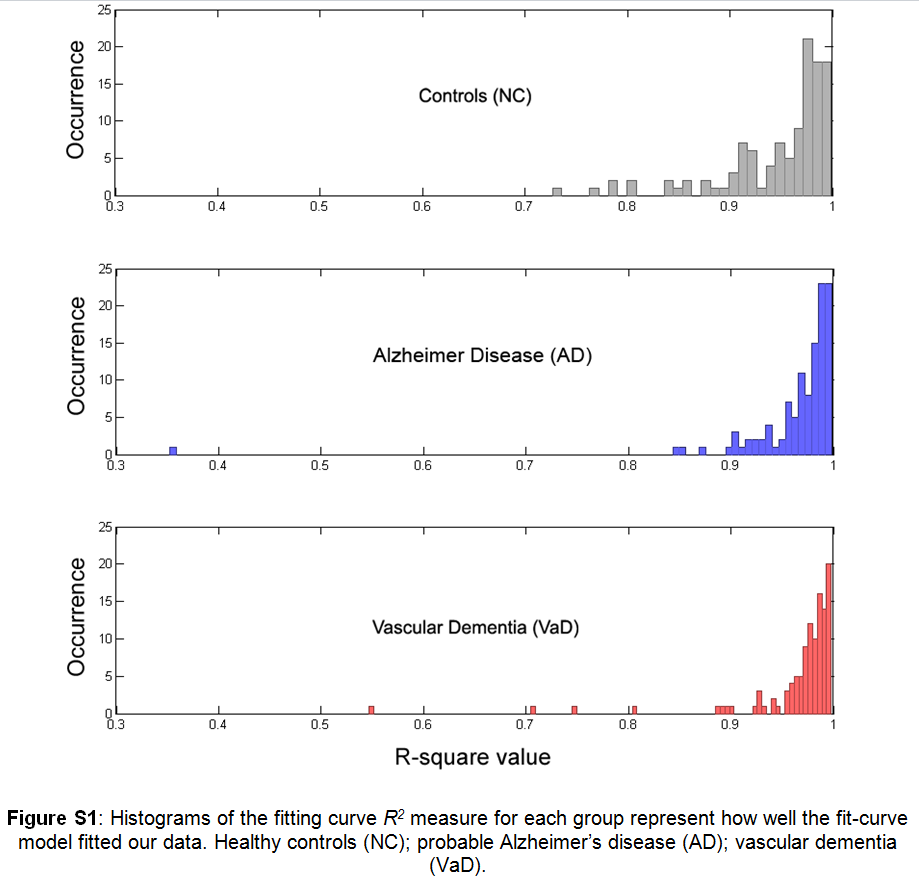

Supplement: Supplementary file 2 [file Image_1.tif]
